# Early brain‐penetrant immunotherapy reverses interferon signature and improves motor outcome in a case of ADAR1‐related Aicardi‐Goutières syndrome

**DOI:** 10.1002/cti2.70113

**Published:** 2026-06-28

**Authors:** Russell C Dale, Jessica Hayes, Velda X Han, Ruwani Dissanayake, Xianzhong Lau, Michelle Farrar, Brian Gloss, Markus Hofer, Esther Tantsis, Michelle Lorentzos, Safiyyah Abbas, Yanick J Crow, Shekeeb Mohammad, Shrujna Patel

**Affiliations:** ^1^ Kids Neuroscience Centre, Faculty of Medicine and Health, The Children's Hospital at Westmead University of Sydney Sydney NSW Australia; ^2^ Faculty of Medicine and Health, The Children's Hospital at Westmead Clinical School University of Sydney Sydney NSW Australia; ^3^ Khoo Teck Puat‐National University Children's Medical Institute National University Health System Singapore Singapore; ^4^ Australian Genome Research Facility Ltd Westmead NSW Australia; ^5^ Australian Genome Research Facility Ltd Melbourne VIC Australia; ^6^ Department of Neurology The Sydney Children's Hospitals Network Sydney NSW Australia; ^7^ Discipline of Paediatrics and Child Health, School of Clinical Medicine, UNSW Medicine and Health University of New South Wales Sydney NSW Australia; ^8^ Westmead Research Hub Westmead Institute for Medical Research Westmead NSW Australia; ^9^ School of Life and Environmental Sciences, Faculty of Science The University of Sydney Sydney NSW Australia; ^10^ Charles Perkins Centre The University of Sydney Sydney NSW Australia; ^11^ MRC Human Genetics Unit, Institute of Genetics and Cancer University of Edinburgh Edinburgh UK; ^12^ Université Paris Cité, Imagine Institute, Laboratory of Neurogenetics and Neuroinflammation INSERM UMR 1163 Paris France

**Keywords:** Aicardi‐Goutières syndrome (AGS), corticosteroid, immune therapy, interferonopathy, transcriptomics

## Abstract

**Objectives:**

Aicardi‐Goutières syndrome (AGS) is a genetic interferonopathy resulting from defects in nucleic acid metabolism and subsequent enhanced type I interferon signalling. We report how an expedited genomic diagnosis in conjunction with natural history data can enable a long‐term brain‐penetrant anti‐inflammatory regimen to optimise neurodevelopmental outcomes in genetic autoinflammatory brain disorders.

**Methods:**

Expedited genomic testing identified compound heterozygous *ADAR1* mutations. Published natural history data from 33 patients with biallelic *ADAR1* mutations reported severe disability (GMFCS V) or death in 79%. To reduce neuroinflammation, we commenced a long‐term pulsed oral dexamethasone protocol (20 mg/m^2^ for 3 days every 3 weeks) from the age of 11 months, plus ruxolitinib, a Janus Kinase (JAK) inhibitor (5 mg per day).

**Results:**

At the age of 24 months, the patient was crawling and walking a few steps unaided, with a GMFCS level of II. Single‐cell RNA sequencing of 41 164 leukocytes, taken before and after 3 months of treatment and compared to three age matched male controls, showed a reversal of upregulated pan‐cellular interferon pathways, with most differentially expressed genes observed in monocytes. On treatment, there was statistically significant downregulation of key autoinflammatory genes, including nucleic acid sensing (*CGAS, IFIH1, SAMHD1*), interferon‐stimulated genes (*ISG15 and IFIF44L*) and signalling (*JAK1*). Given the dual immune therapy, it was not possible to define whether the biological effect was related to dexamethasone or JAK inhibitor, or both.

**Conclusion:**

Compared with natural history data, our data suggest that early diagnosis, and the use of early brain‐penetrant immune suppressants (dexamethasone), may improve outcomes in *ADAR1* AGS.

## Introduction

**Figure 1:**
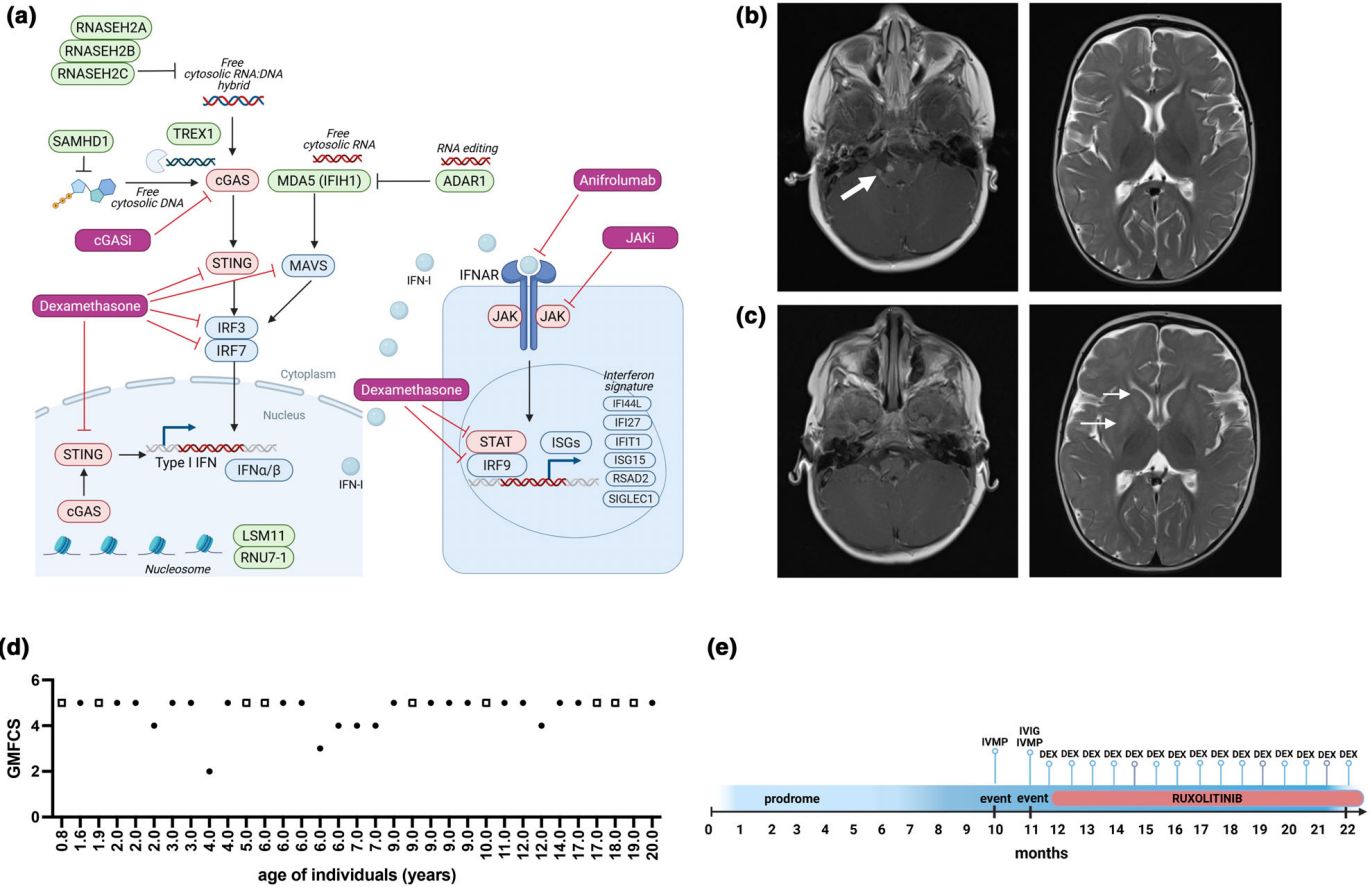
**(a)** RNA or DNA sensing through the MAVS or cGAS‐STING pathways results in activation of interferon transcription factors (IRF3 and IRF7), and type I interferon production. Type I interferon (IFN‐I) binds to the interferon receptor and activates the Janus Kinase (JAK)–STAT signalling pathway and further interferon gene activation. cGAS inhibitors (cGASi) offer the possibility for targeted therapeutics in cases of Aicardi‐Goutières syndrome (AGS) induced by DNA species, while JAK inhibitors (JAKi) and anifrolumab target interferon signalling downstream. Corticosteroids such as dexamethasone have inhibitory effects on many transcription factors and adaptor proteins, including STING, MAVS, IRFs and STAT. AGS genes are in green, and the CGAS‐STING and JAK–STAT genes in pink. **(b, c)** During first presentation, there was a well circumscribed lesion in the medulla (**b**, left), which resolved during second episode (**c**, left). At the second episode, there was hyperintensity in the caudate and putamen bilaterally (**c**, right). **(d)** The GMFCS scores presented by age at last follow‐up in patients harbouring biallelic mutations in *ADAR1* as described by Rice et al. Each symbol represents an individual patient: black circle for alive patients at last follow‐up, white square for patients who had died. **(e)** Clinical course and therapeutics in our case.

Described initially by Jean Aicardi and Francoise Goutières as a familial syndrome of early onset neuroregression, cerebrospinal fluid (CSF) pleocytosis and intracranial calcification, the subsequent discovery of mutations in any one of nine causative genes has redefined Aicardi‐Goutières syndrome (AGS) as a genetic interferonopathy because of aberrant metabolism of DNA and RNA, leading to a type I interferon neuroinflammatory response.^1^ AGS most frequently involves the brain and skin, and peripheral immune cells manifest persistent upregulation of type I interferon signalling. Despite a major improvement in the understanding of underlying disease pathology, the treatment of the neurological disorder remains limited, with high morbidity and mortality. JAK inhibitors (e.g. baricitinib or ruxolitinib) and type 1 interferon receptor antagonists (e.g. anifrolumab), both acting on pathways downstream of the defects in DNA and RNA metabolism (Figure 1a), show some promise but have poor brain penetration, which may limit their clinical efficacy in AGS.^2^ We present a patient in whom early diagnostic testing facilitated the initiation of a long‐term immune therapy protocol using dexamethasone pulses, a drug with excellent brain penetration, in conjunction with ruxolitinib.

## Results

### Case history

A neurodevelopmentally normal 9‐month‐old male presented with a 24‐h history of motor decline. He has a neurodevelopmentally normal 4‐year‐old sister, and both parents are well and unrelated. Before the illness, he was able to sit, roll, stand with support and babble. In the 3 weeks before his presentation, he had an *Escherichia coli* urinary tract infection and a subsequent enteroviral infection (confirmed on nasopharyngeal aspirate).

The acute illness was characterised by loss of truncal tone, increased tone in the limbs with brisk reflexes, loss of the ability to sit, loss of the ability to babble, tongue tremor and two focal seizures. CSF examination revealed no pleocytosis, negative oligoclonal bands and negative viral testing. However, CSF neopterin was raised to 4.6× the upper limit of normal (138 nmoL/L, normal < 30). Cranial MRI revealed a well circumscribed T2 hyperintense and T1 hypointense lesion in the right ventral medulla of the brainstem (Figure 1b) and normal basal ganglia. He was treated as a case of suspected encephalitis, with acyclovir, antibiotics, levetiracetam and 3 days of 30 mg/kg of methylprednisolone. After a 9‐day admission and partial improvement in his motor function, he was discharged home.

Four weeks after his first event, he experienced a second episode, with a further deterioration and loss of acquired skills, floppiness with generalised weakness, but with preserved social engagement and eye contact. MRI brain showed near complete resolution of the brainstem lesion, but new symmetrical hyperintensities of the basal ganglia (caudate and putamen) (Figure 1c). Repeat CSF again showed no pleocytosis, with reduced, but still mildly elevated, CSF neopterin (42 nmoL/L). He was given a further course of intravenous methylprednisolone, plus 2 g/kg of intravenous immunoglobulin, followed by prednisolone 10 mg once per day. An urgent trio exome identified compound heterozygous variants in *ADAR1* (both pathogenic): c.577C>G p.(Pro193Ala) and c.1747G>T p.(Glu583*), with appropriate parental segregation. He has not manifested any skin or multi‐organ involvement (including a normal echocardiogram).

For context, we analysed the natural history data of 33 patients with biallelic *ADAR1* variants extracted from Rice et al.^3^ (G1007R dominant negative patients excluded) (Figure 1d). The GMFCS score showed that 26 of 33 (79%) patients had GMFCS V (*n* = 17) or had died (*n* = 9), four patients had GMFCS IV, and one patient each had GMFCS III and GMFCS II (age at final follow‐up in Figure 1d). No patients had a score of GMFCS I.

In view of the relapsing autoinflammatory brain condition and concerning natural history, we commenced the patient on 20 mg/m^2^ dexamethasone per day (6 mg twice per day) for 3 days, every 3 weeks, commencing 4 weeks after his second event, and prednisolone was ceased (Figure 1e). One month later, at the age of 12 months, ruxolitinib 5 mg once per day was commenced. He subsequently received intensive physiotherapy, long‐term steroid monitoring which includes six‐monthly eye, bone, growth and metabolic monitoring, and a long‐term immune suppression safety plan.

By the age of 13 months, the child had regained some motor skills, being able to sit again and to stand with support, while manifesting evolving dystonic posturing. By the age of 15 months, he could commando crawl; the family noticing that he could crawl across the room more ably in the 2 weeks after a dexamethasone pulse, while in the week before steroids were due, he could only make a few crawling movements. At this age, he knew the names of 20 objects, could point, was vocal and had good eye contact and social intent.

By the age of 19 months, he could crawl at speed, stand against furniture and use a modified pincer grip. By the age of 22 months, he was able to take steps unaided. He had evolving dystonia and spasticity with a left‐handed preference. At the age of 24 months, he was graded at GMFCS II (walks with limitations, may struggle with balance) (Supplementary video 1). He remains on ruxolitinib 5 mg per day and dexamethasone pulses (currently 8 mg twice a day for 3 days, every 3 weeks). The dexamethasone regimen has been tolerated without significant cushingoid effects, although he is irritable for the second and third day of the dexamethasone pulse. MRI brain has not been repeated yet.

### Single‐cell RNA sequencing of peripheral blood

**Figure 2:**
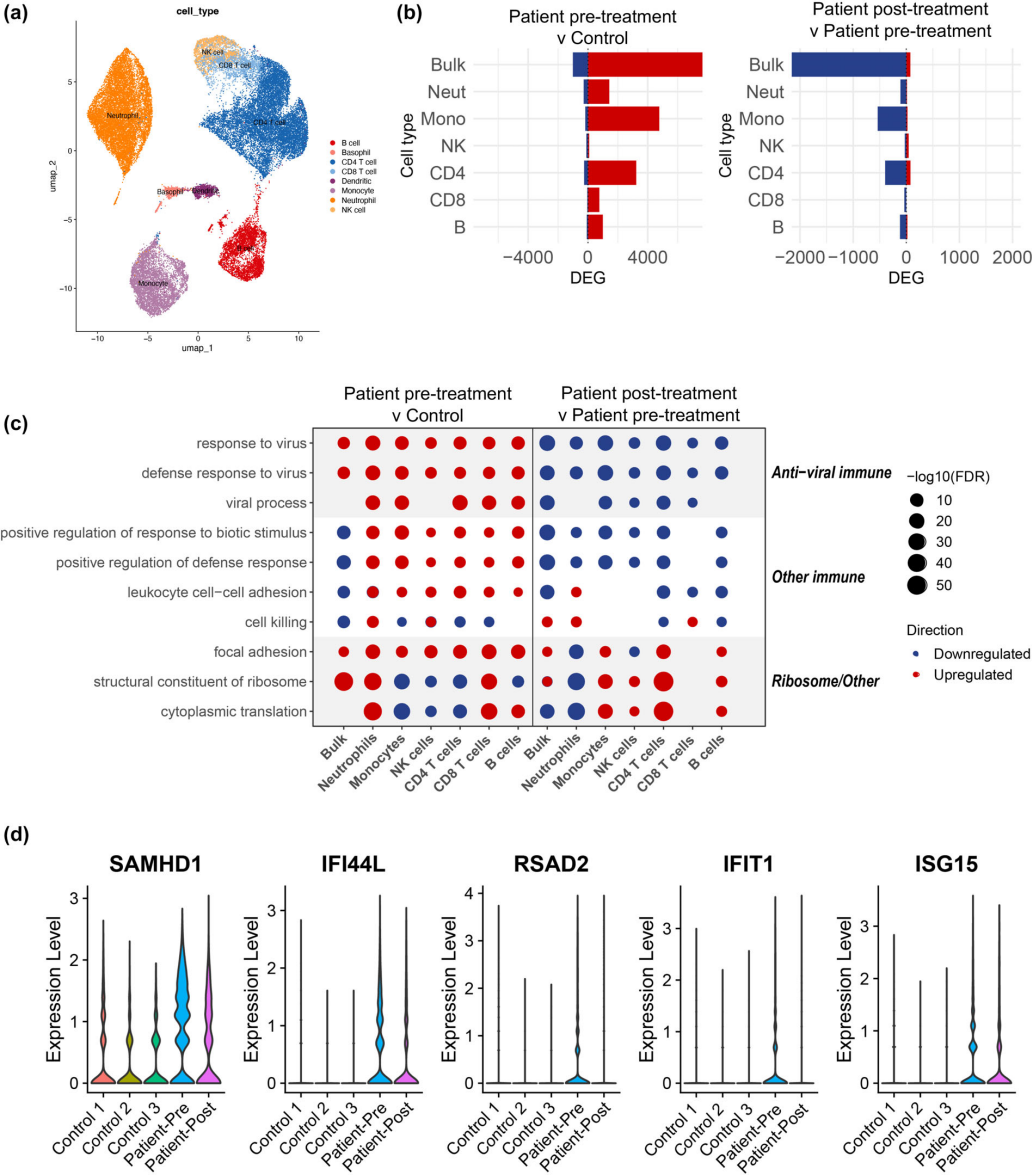
**(a)** UMAP reveals the cell clusters in patient and control cells (total 41 164 cells). **(b)** Up‐ (red) and downregulated (blue) differentially expressed genes (DEGs) [false discovery rate (FDR) < 0.05] are presented by cell type at baseline (pre vs control) on left, and after treatment (post vs pre) on right. Monocytes and CD4 cells had the most DEGs in both comparisons. **(c)** Bulk (all cells) and the six main cell types reveal the most significant pathways at baseline (pre vs control) and after treatment (post vs pre). Only pathways present in five or more cell types are presented. Viral pathways are upregulated at baseline (red) and downregulated by treatment (blue). **(d)** Bulk gene expression for *SAMHD1*, *IFI44L*, *RSAD2*, *IFIT1* and *ISG15* are presented in the three controls and the patient pre‐ and post‐treatment. Gene expression was elevated in patient‐pre vs controls (adj. *P*‐values all < 10^−140^), and the patient‐post versus patient‐pre (adj. *P*‐values all < 10^−35^). In patient‐post, the gene expression approaches control values for *RSAD2* and *IFIT1*, whereas for *SAMHD1*, *IFI44L* and *ISG15*, the patient‐post values were reduced but still elevated.

From the two patient samples (pre‐dexamethasone pulse at 11 months and on dexamethasone and ruxolitinib treatment at 14 months) and three controls, a total of 41 164 cells were sequenced, encompassing eight main cell types (Figure 2a), with our subsequent analysis focused on neutrophils, monocytes, natural killer, CD4+ T cells, CD8+ T cells and B cells. The numbers of differentially expressed genes (DEGs) (FDR < 0.05) are presented in Figure 2b. There were more upregulated DEGs at baseline (pre vs controls), but more downregulated DEGs in the post‐ versus pre‐comparison, with monocytes revealing the largest number of DEGs in both comparisons (Figure 2b). We focused on the most common findings across cell types (significant pathways in five or more cell types, Figure 2c).

At baseline, there was upregulation of the ‘response to virus’ pathway and other antiviral innate immune pathways across all cell types, and these pathways were significantly downregulated following 3 months of treatment (Figure 2c, pathway adjusted *P*‐value of 10^−5^ to 10^−50^). Other immune pathways were also significantly dysregulated (generally, but not uniformly, upregulated), which were also downregulated by treatment. Ribosomal pathways were dysregulated at baseline and were generally reversed with treatment (Figure 2c).

### Response to virus pathway

Focusing on the ‘response to virus’ pathway, in the bulk comparison, 81 genes were significantly upregulated at baseline and significantly downregulated by treatment. The functions of these genes included nucleic acid sensing (e.g. *CGAS, IFIH1* and *SAMHD1*), RNA processing, interferon‐stimulated genes (e.g. *IFI44L and ISG15*) and signalling genes (e.g. *JAK1*) (Table 1). The gene expression values of selected type 1 interferon‐related genes (*IFI44L, RSAD2, IFIT1 and ISG15*) and *SAMHD1* in this pathway are presented in Figure 2d.

**Table 1:** Overlapping genes from ‘Response to virus’ pathway: In bulk analysis, 81 genes were significantly upregulated [false discovery rate (FDR) < 0.05] at baseline (patient baseline vs controls) and significantly downregulated (FDR < 0.05) by treatment (patient‐treated vs patient baseline)

Category	Genes	Key function
1. Nucleic acid sensing	*CGAS, IFIH1, DDX60, DHX58, DHX36, DHX9, DDX1, DDX21, TLR7, UNC93B1, SAMHD1*	Viral RNA/DNA detection and upstream innate sensing
2. RNA processing/metabolism	*DHX15, CNOT7, HNRNPUL1, PCBP2, LSM14A, NT5C2, NT5C3A, ZC3HAV1*	RNA stability, decay, antiviral RNA handling
3. Interferon‐stimulated genes (ISGs)	*ISG15, IFI44, IFI44L, IFIT1, IFIT2, IFIT5, OAS1, OAS2, OAS3, OASL, MX1, RSAD2, BST2, GBP1, HERC5, PARP9, TRIM5, TRIM56*	Direct antiviral restriction and interferon effector response
4. Innate immune effectors	*AZI2, FGL2, LILRB1, SPN, LGALS8, NPC2, SERINC3, SERINC5, RNF216, C1QBP, CD2AP*	Immune activation, pathogen restriction, host defence
5. Signalling (non‐transcriptional)	*JAK1, TGFB1, PIM2, CDK6, DTX3L, TOMM70, UBE2N*	Signal propagation downstream of immune receptors
6. Transcription factors/regulators	*STAT1, STAT2, NFKB1, RELA, IRF7, GATA3, POU2F2, SIN3A, TRIM44, SENP7*	Interferon signalling, NF‐κB activation, transcriptional control
7. Autophagy/cellular stress/apoptosis	*BECN1, BNIP3L, PHB2, PMAIP1, HSP90AA1, TPT1, G3BP1, CFL1, ENO1, BCL2*	Stress response, mitochondrial quality control, apoptosis
8. Translation/proteostasis	*EIF2AK2, EIF5A, EEF1G, PSMA2, SEC11A, URI1*	Translational control and protein turnover

The 81 genes were clustered according to function.

## Discussion

In our case, the fluctuating phenotype and expedited gene testing allowed for a rapid diagnosis of AGS. This provided an opportunity to implement a long‐term neuroinflammatory therapy protocol without major interruptions in treatment, with the interval between steroid pulses never more than 4 weeks. In the largest cohort of *ADAR1* cases yet published (*n* = 46), Rice et al. described a number of clinical phenotypic groups, including ‘classical AGS’, bilateral striatal necrosis, spastic paraplegia and a progressive motor syndrome.^3^ Over half of the patients had apparent normal development prior to onset, and an infectious trigger was noted in a significant minority. The onset was abrupt in some, but more progressive over weeks or months in others.^3^ A fluctuating course was not described in the Rice et al. cohort: we hypothesise that the phenotype in our patient may have been due to the use of high dose corticosteroid during the first event, with a subsequent episode when the steroid pulse had lost its effect at 4 weeks.

*ADAR1* is involved in the editing of double stranded RNA (dsRNA), with sensing of unedited dsRNA via the MAVS adaptor protein leading to activation of type I interferon signalling (Figure 1). *ADAR1* is expressed in all nuclei containing cells, with high expression in neurons, glia and immune cells. In AGS, there is higher type I interferon production in the brain vis a vis the blood^4^; therefore, drugs with high brain penetrance may be required to treat the neurological features of AGS. JAK inhibitors, which have poor brain penetration, have biological effect on the peripheral interferon signature in AGS, but a more modest effect on the neurological disease.^1^, ^5^, ^6^ Likewise, anifrolumab, a type 1 interferon receptor antagonist, like all monoclonal antibodies, has poor penetration of CNS.^7^ By contrast, corticosteroids have excellent brain penetration. We used the ‘Pranzatelli pulse’ regimen, as used in opsoclonus myoclonus ataxia syndrome,^8^ another infantile chronic inflammatory brain condition, for which dexamethasone pulses are used for months and years with improved outcomes. Used every 3 weeks, our experience suggests that there are less cushingoid effects compared to daily glucocorticoids with this regimen. The risk of doing nothing in *ADAR1‐*related AGS, as outlined in Figure 1d, is high, with significant associated morbidity and mortality. The steroid protocol employed here is not without risk of infection, and impacts on growth and bone mineralisation. However, we suggest that corticosteroid may offer hope for patients until better brain‐penetrant drugs are found. *PTPN1* haploinsufficiency can cause a subacute encephalopathy in childhood associated with upregulated interferon‐stimulated genes, and corticosteroids has been reported to be of benefit in affected patients.^9^, ^10^ The previous literature reporting steroid use in AGS is limited to small numbers and variable clinical scenarios.^11^, ^12^, ^13^

The scRNA‐seq provided biological evidence that the drug regimen used here was able to reverse the peripheral immune interferon signature. scRNA‐seq is a powerful statistical tool, allowing the examination of gene expression of thousands of patient peripheral immune cells compared to control cells. The dysregulated genes at baseline and after treatment included genes known to be central to AGS biology (such as *SAMHD1, CGAS and IFIH1*), four of the six interferon‐stimulated genes used in a standard ISG panel (*ISG15, IFI44L, RSAD2 and IFIT1*), and key signalling genes (*JAK1*). We have employed similar *ex vivo* scRNA‐seq approaches to define the effects of drugs in other neuroinflammatory settings.^14^, ^15^ There were similar upregulated inflammatory pathways across all cell types, noting that some interferon producing cell types, such as plasmacytoid dendritic cells, were too rare for adequate detection and analysis.

The question remains as to how long, such a regimen be continued. Our hypothesis is that the neuroinflammatory environment negatively affects neurodevelopment so that we intend to continue our protocol into the fourth year of life, subject to safety monitoring. Previous data have shown that the neuroinflammation in AGS is most active in the first months and years after disease presentation and reduces over time.^16^ In addition, corticosteroid cessation can be associated with neuroinflammatory flares in AGS. We believe the dexamethasone pulses mitigate the risk by avoiding sustained continuous exposure and abrupt cessation. The plan will be to begin tapering dexamethasone pulses after the fourth birthday: we intend to maintain the same dose until age 4, and assuming stable, start tapering the dose while maintaining the three weekly interval, to 6 mg twice per day for 3 days for four cycles, then 4 mg twice per day for 3 days for four cycles, then 2 mg twice per day for 3 days for two cycles, then 2 mg once per day for 3 days for two cycles, then cease. Throughout this taper, there will be attention to any decline in function, particularly in the week before the pulse is due. The JAK inhibitor will be continued throughout this wean and continue for at least two further years after cessation of corticosteroids.

The limitations of our report are the fact that it involves a single case, the unknown natural history in this specific case with these specific compound heterozygous variants, uncertainty as to whether a similar approach would be useful in other AGS patients, and the lack of serial neuroimaging and longer‐term outcome. In addition, we do not know whether the clinical and biological effect (as shown with scRNA‐seq) was related to dexamethasone or JAK inhibitors, or both. However, we propose that this ‘early combined intervention’ was associated with a better outcome than expected, in this patient. However, given the otherwise dire expected clinical outcome, we believe that this case provides an example of how using natural history data, disease biomarkers and open label therapeutics holds promise in the *n* = 1 rare disease context and provides some evidence that early diagnosis and intervention can improve outcomes in AGS.

## Method

We report a case of AGS presenting with relapsing encephalopathy and bilateral striatal necrosis due to compound heterozygous *ADAR1* mutations. The relapsing presentation resulted in expedited genomic testing and institution of a long‐term neurotherapeutic protocol.

To assess the outcome in our case, we used data from the largest published *ADAR1* cohort to generate ‘natural history outcome’ data of patients with biallelic *ADAR1* (compound heterozygous or homozygous) in the JAKi pre‐treatment era.^3^ Patients with the p.(G1007R) heterozygous state were excluded. We used the gross motor function classification system (GMFCS), which ranges from Level I (walks without limitations) to Level V (transported in a manual wheelchair, severely limited ability to maintain posture or move independently), as reported by Rice et al.^3^

Single‐cell RNA sequencing (scRNA‐seq) of peripheral blood was used to explore immune cell dysfunction and drug effects. We used the HIVE™ Single Cell platform, and whole leukocytes from the patient before starting treatment (at the age of 11 months), and after 3 months of treatment (at the age of 14 months). We compared the findings in our patient with the results of three healthy neurodevelopmentally normal infant boys (the age of 12–24 months). scRNA‐seq data were analysed in the R statistical environment with *tidyverse*, as previously described, using the *Seurat* package for analysis.^14^ Pathway enrichment analysis was performed via over‐representation analysis (ORA) to obtain enriched Gene Ontology (GO) pathways [false discovery rate (FDR) < 0.05] using the *clusterProfiler* package.^14^

Ethics approval was granted by the Sydney Children's Hospitals Network Human Research Ethics Committee (2021/ETH00356).

## Author contributions

**Russell C Dale:** Conceptualization; investigation; funding acquisition; writing – original draft; methodology; writing – review and editing; supervision; resources. **Michelle Farrar:** Writing – review and editing; supervision; funding acquisition. **Velda X Han:** Writing – review and editing; methodology; supervision. **Ruwani Dissanayake:** Writing – review and editing; methodology; data curation. **Jessica Hayes:** Investigation; writing – review and editing; methodology; project administration. **Safiyyah Abbas:** Investigation; writing – review and editing. **Shrujna Patel:** Writing – original draft; writing – review and editing; methodology; visualization; formal analysis; project administration; supervision; data curation. **Xianzhong Lau:** Methodology; writing – review and editing; data curation. **Yanick J Crow:** Conceptualization; investigation; supervision; writing – review and editing. **Shekeeb Mohammad:** Investigation; writing – review and editing; supervision. **Esther Tantsis:** Writing – review and editing; supervision; investigation. **Markus Hofer:** Writing – review and editing; methodology; supervision. **Brian Gloss:** Formal analysis; software; data curation; methodology; visualization. **Michelle Lorentzos:** Writing – review and editing; supervision; investigation.

## Conflict of interest

The authors declare no conflict of interest.

## Supporting information


Supplementary video 1

## Data Availability

The data that support the findings of this study are available from the corresponding author upon reasonable request.
